# Cyber victimisation and depression among adolescents in Tunisia: a case report study and review of literature

**DOI:** 10.1192/j.eurpsy.2024.932

**Published:** 2024-08-27

**Authors:** E. Bergaoui, A. Touiti, A. Maamri, A. Hajri, H. Zalila

**Affiliations:** ^1^Faculty of medicine of Tunis, University Tunis Manar, Manar; ^2^Oupatient psychiatry department, Razi hospital, Manouba, Tunisia

## Abstract

**Introduction:**

Cyber victimization is a form of violence that is perpetrated through social media, and its victims are primarily adolescents and young adults. This can have a negative impact on their psychosocial well-being.

**Objectives:**

To investigate the relationship between cyber victimization, depression, and suicide, identifying risk factors, prevention and intervention strategies through an case report.

**Methods:**

We report the clinical case of a 16-year-old Tunisian man who developed a depressive disorder after being cyber-victimized. We also conducted a literature review in PubMed database keywords: depression, suicide, cybervictimisation, adolescents to identify risk factors, prevention and intervention strategies.

**Results:**

The adolescent was a member of a youth group called The Gung, which organized climbing challenges that were then broadcast on Facebook. He was the victim of cyberbullying after failing a challenge that was broadcast live. As a result, he was rejected by his group of friends and subjected to death threats and bullying. A clinical examination revealed major depressive disorder, low self-esteem, and low self-assertion. The patient was treated with a combination of medication and psychotherapy, and he had a good outcome with social and educational reintegration.

Several studies have found that cyber victimization is associated with depressive disorders, anxiety disorders, and suicidal behavior among youth. Several risk factors have been identified, including low socioeconomic status, disrupted family dynamics, low self-esteem, and psychiatric disorders. Prevention and intervention strategies involve families, educational institutions, civil society, and health professionals.

**Conclusions:**

The seriousness of cyber victimization among youth is undeniable. Early and personalized intervention is necessary to prevent suicidal behavior and restore the well-being of adolescents.

**Disclosure of Interest:**

None Declared

